# Differences between CusA and AcrB Crystallisation Highlighted by Protein Flexibility

**DOI:** 10.1371/journal.pone.0006214

**Published:** 2009-07-10

**Authors:** Aurélien Deniaud, Aurélie Goulielmakis, Jacques Covès, Eva Pebay-Peyroula

**Affiliations:** Institut de Biologie Structurale Jean Pierre Ebel, UMR5075 CEA-CNRS-Université Joseph Fourier, Grenoble, France; Griffith University, Australia

## Abstract

**Background:**

Until very recently, AcrB was the only Resistance Nodulation and cell Division transporter for which the structure has been elucidated. Towards a general understanding of this protein family, CusA and AcrB were compared.

**Methodology/Principal Findings:**

In dodecylmaltoside, AcrB crystallised in many different conditions, while CusA does not. This could be due to the difference in dynamic between these proteins as judged from limited proteolysis assays. Addition of various compounds, in particular heavy metal cations, stabilises CusA.

**Conclusion/Significance:**

This approach could constitute a first step towards CusA crystallisation.

## Introduction

As any gram-negative bacteria, *Escherichia coli* possesses several multicomponent transporters of the Resistance, Nodulation and cell Division (RND) family responsible for drugs (HAE-RND) and heavy metal export (HME-RND) [1], [2]. This inner membrane protein is part of a tripartite protein complex together with a periplasmic membrane fusion protein (MFP) and an outer membrane factor (OMF) [3]. The export of toxic compounds is driven by proton import catalyzed by the RND proteins [4]. *E. coli* contains only one member of HME-RND family, CusA, which confers copper and silver resistance [5] to the bacteria.

**Figure 1:**
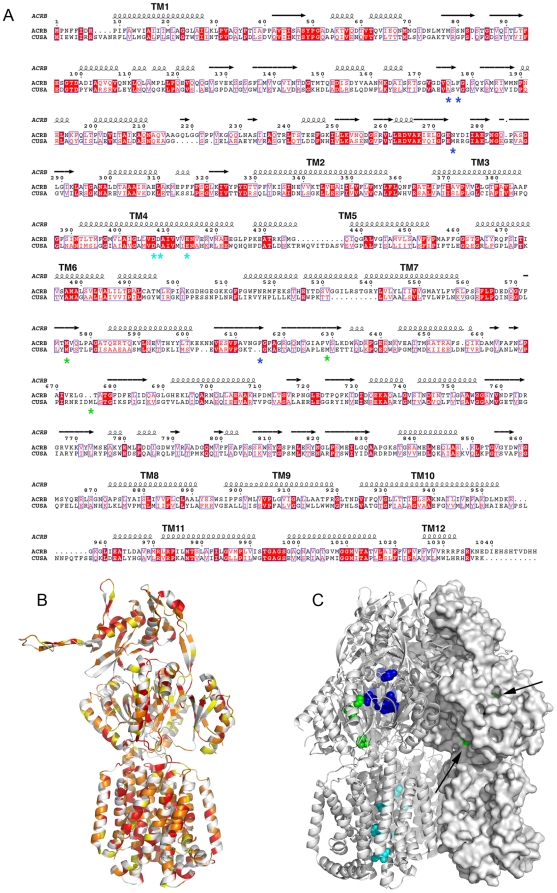
Sequence alignments of CusA with AcrB. Panel A, the sequence of CusA was compared to AcrB. The figure was prepared with ESPript (http://espript/ibcp.fr). The secondary structure is indicated above the sequence according to the AcrB structure (PDB code 1IWG). Residues known to be involved in proton translocation are labelled with cyan stars. AcrB residues implicated in ligand binding are highlighted by blue stars. CusA residues important for the copper resistance are shown as green stars. Panel B, the ribbon representation of the AcrB monomer. Residues are coloured red, orange, yellow or white according to the comparison with CusA (red for identical, white for non conserved, and orange and yellow for similar residues). Panel C, the ribbon representation of the AcrB trimer. Residues implicated in proton translocation and ligand binding are shown as spheres in cyan and blue, respectively. Residues that are equivalent to M573, M623 and M672 of CusA are depicted as spheres in green. The surface representation of the monomer to the right highlights the accessibility of these residues from the periplasm (shown by arrows).

AcrB structure has been solved to quasi-atomic resolution [6], [7], but no HME-RND structure has been elucidated so far. Average sequence identity is around 60–70% in each sub-family and 20% between the two. The sequence alignments of AcrB and CusA illustrate the divergence between the two sub-families (fig. 1A). Moreover, the differences and similarities between CusA and AcrB are highlighted on the structure of AcrB (fig. 1B–C). These two panels reveal that the inner core of the transporters is the most conserved part and this is particularly true for the transmembrane domain. The RND signature, located in the fourth transmembrane helix and comprising several acidic aminocids around residue 400, is almost conserved between the HME and HAE sub-families (Figure 1A and 1C). This sequence is essential for proton translocation for AcrB [8], [9] and CusA. Indeed, the mutations of D405 and E412 in CusA affect the function of the transporter as shown by the loss of copper resistance [5]. In contrast with the RND signature, the residues implicated in ligand binding for AcrB and copper resistance for CusA are located in different sites (fig. 1). The three methionines of CusA described to be important for copper resistance are located in the second large periplasmic domain [5].

The work published by Stroebel *et al.* [10] constituted a first step towards an explanation for CusA reluctance to crystallisation. The authors compared CusA and AcrB by analytical ultracentrifugation and infra-red spectroscopy. The oligomeric state of CusA and AcrB in dodecyl-β-D-maltopyranoside (C_12_M) appeared pretty similar. Stroebel *et al.* also observed that CusA, contrary to AcrB, retains some lipids after purification in C_12_M. In the present study, crystallogenesis and flexibility of these proteins were compared. Preparation of a rigid form, *i.e.* a specifically locked conformation, is a prerequisite for protein crystallisation. Indeed, compact proteins are well-defined three-dimensional objects, and therefore favour protein-protein contacts necessary for crystallisation. Limited proteolysis is a common tool to identify flexible loops in proteins that could prevent appropriate crystal packing [11], [12], or to determine conditions to obtain a rigid form of the protein. A completely opposite behaviour between AcrB and CusA in terms of conformational states explored was clearly observed. Thus, AcrB has a very rigid core while CusA seems highly flexible. We describe a strategy to prepare the HME-RND protein, CusA, with a flexibility reduced by the addition of heavy metal cations. We propose that this might open the way towards CusA crystallisation.

## Results

### AcrB, CusA crystallisation

**Figure 2:**
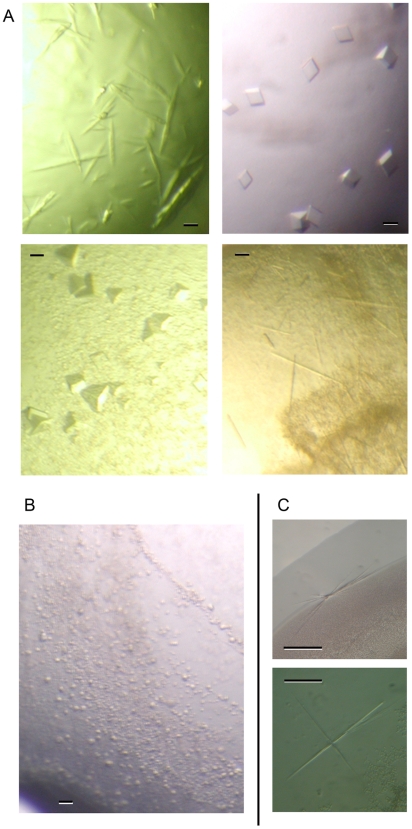
Comparison of AcrB and CusA crystallisation. Panel A, AcrB crystals obtained in four conditions of initial screens in nanodrop assays. Panel B, small granules obtained with CusA in C_12_M in initial nanodrop screens. Panel C, needles and bunches of needles obtained with CusA in C_12_E_8_. In all panels, scale bar corresponds to 100 µm.

AcrB in C_12_M crystallised in 5% of the initial 1200 conditions of commercial and membrane protein optimised home-made screens tested (a few examples are shown in fig. 2A), while no crystals could be detected for CusA in the same detergent. However, several interesting objects were obtained with small PEGs as precipitant, and MgCl_2_ as additive (fig. 2B). Optimisation of these conditions did not lead to crystals of CusA purified in C_12_M. CusA was then purified in 13 other detergents (table 1) and for each detergent, 192 crystallisation conditions derived from the initial interesting conditions in C_12_M were tested (table 1). In this detergent screen, very thin needles and bunches of needles were obtained in C_12_E_8_ (fig. 2C). However, these crystals did not show any protein diffraction pattern (not shown).

**Table 1:** Different detergents screened for CusA crystallisation.

Detergent	Used concentration	Detergent properties	Maximal protein concentration	Crystallisation observations
β-dodecylmaltoside	0.04%	N, cmc = 0.0087%	10 mg/ml	not well-defined plates and rods
β-dodecylthiomaltoside	0.01%	N, cmc = 0.0026%	5 mg/ml	-
β-undecylmaltoside	0.12%	N, cmc = 0.029%	10 mg/ml	not well-defined plates and rods
α-dodecylmaltoside	0.03%	N, cmc = 0.0076%	8–10 mg/ml	-
cymal-6	0.11%	N, cmc = 0.028%	8–10 mg/ml	not well-defined plates and rods
C_12_DAO	0.09%	Z, cmc = 0.023%	at least 10 mg/ml	nice precipitates
C_10_DAO	0.84%	Z, cmc = 0.21%	10 mg/ml	nice precipitates
C_12_E_9_	0.01%	N, cmc = 0.003%	at least 15 mg/ml	-
C_12_E_8_	0.02%	N, cmc = 0.0048%	50 mg/ml	needles, bunches of needles and platelets
C_10_E_5_	0.12%	N, cmc = 0.031%	at least 10 mg/ml	-
C_8_E_6_	1.56%	N, cmc = 0.39%	2 mg/ml	-
C_8_E_4_	1%	N, cmc = 0.25%	3 mg/ml	very small bunches of needles
β-octylglucoside	2.12%	N, cmc = 0.53%	1–2 mg/ml	-
Fos-choline12	0.19%	Z, cmc = 0.047%	protein lost on streptactin column during buffer exchange	no crystallisation assay

Maximal concentration obtained at the end of CusA purifications and results observed with the 192 crystallisation conditions tested for each detergent assayed. N = non-ionic detergent, Z = zwitterionic detergent.

### Comparison of AcrB and CusA dynamic

The dynamical behavior of CusA and AcrB purified in C_12_M was compared by limited proteolysis. Six different proteases were tested: trypsin, chymotrypsin, elastase, subtilysin, papain and thermolysin, at different protease to protein weight ratios ranging from 1∶200 to 1∶10000. Only representative experiments are presented in the figures.

**Figure 3:**
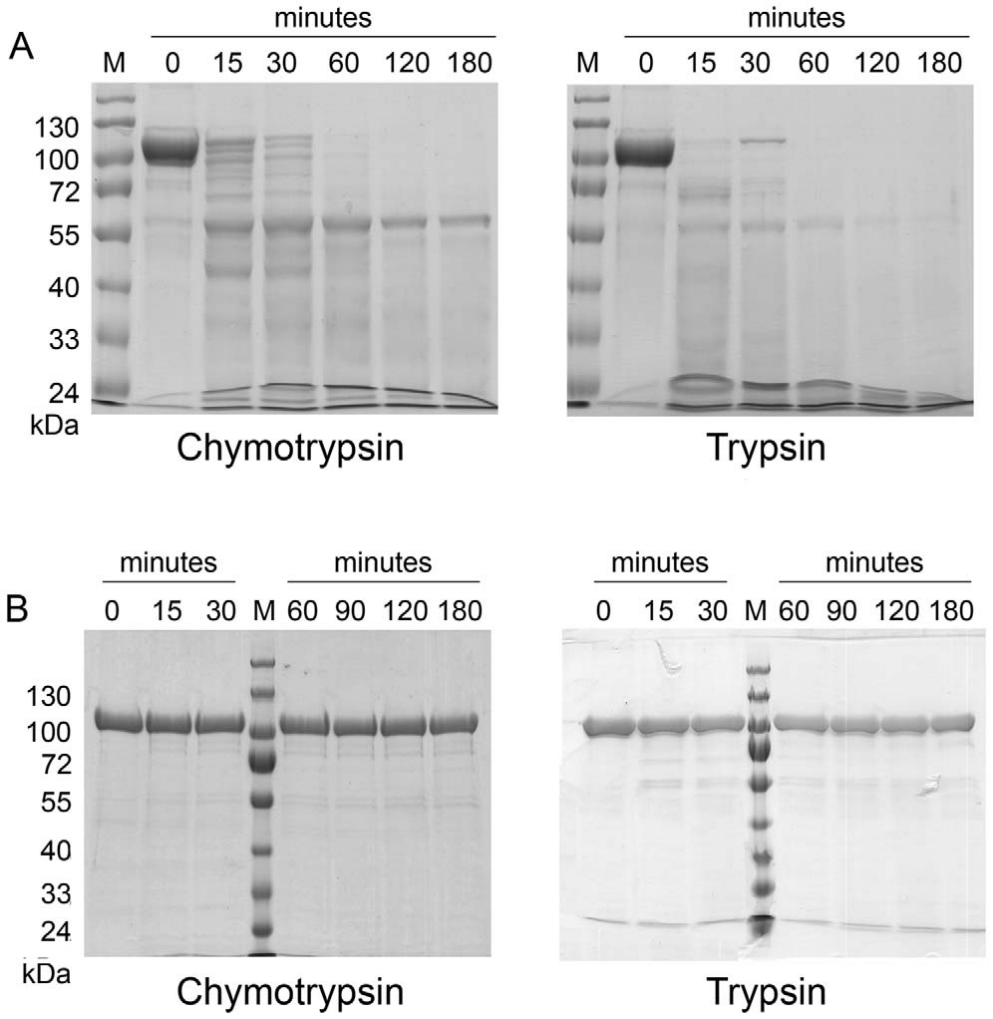
Comparison of AcrB and CusA limited proteolysis. Panel A, proteolysis kinetics of CusA in C_12_M at a 1∶1000 ratio. Panel B, proteolysis kinetics of AcrB in C_12_M at a 1∶1000 ratio. M = Molecular weight markers.

Full-length CusA was totally proteolysed in less than 15 minutes with three proteases: trypsin (fig. 3A), papain and subtilysin. Trypsin cleaved CusA into several unstable fragments ranging from 20 to 70 kDa. Chymotrypsin (fig. 3A), elastase and thermolysin allowed the release of several fragments ranging from 20 to 80 kDa and stable during 15 to 180 minutes. One band around 65 kDa seemed particularly stable and appeared similar for these three proteases. In all cases the whole protein was not stable for more than thirty minutes to one hour. The high number of short-life fragments obtained with chymotrypsin, thermolysin and elastase prevented the purification and the exact identification of CusA rigid domains.

While subtilisin and papain proteolysed AcrB at a 1∶1000 ratio, trypsin (fig. 3B), chymotrypsin (fig. 3B), elastase and thermolysin were inefficient at this ratio. Even at a 1∶200 protease:protein ratio, no or little proteolysis was observed with elastase and thermolysin (not shown).

### CusA stabilisation by amphiphiles or heavy metal additives

To reduce CusA dynamic two strategies were considered: the use of various additives *i.e.* different amphiphiles or the addition of CusA ligands such as metals, which are putative substrates of this inner membrane transporter.

#### Amphiphile strategy

**Figure 4:**
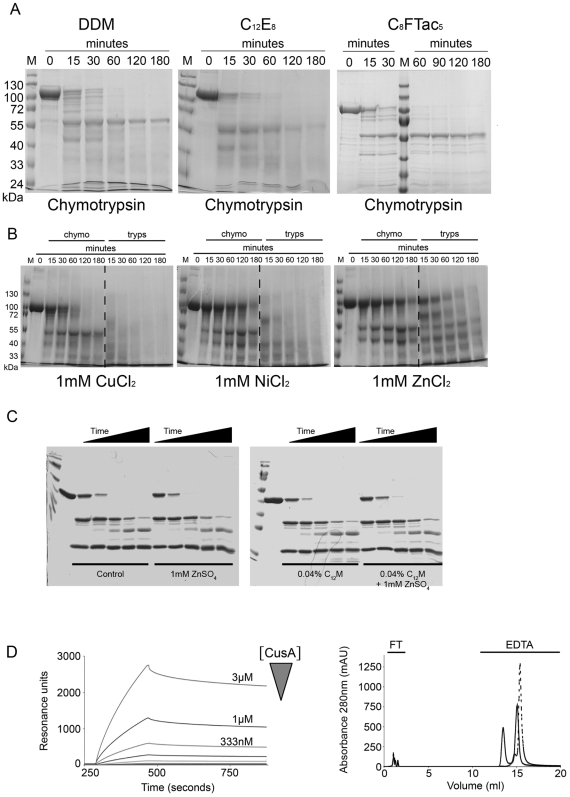
Effect of various additives on CusA. Panel A, chymotrypsinolysis kinetics of CusA in C_12_M, C_12_E_8_, and C_8_FTac_5_. Panel B, proteolysis kinetics of CusA in C_12_M in the presence of different heavy metal cations. Panel C, Chymotrypsinolysis kinetics of p47phox in purification buffer alone, purification buffer with 1 mM ZnSO_4_, purification buffer with 0.04% C_12_M or purification buffer with 1 mM ZnSO_4_ and 0.04% C_12_M. Panel D, left graph corresponds to SPR measurements, dose-response double-subtracted curves of CusA in C_12_M binding on a Ni-NTA flow cell. Increasing concentrations of CusA are: 1.4 nM, 4.1 nM, 12.3 nM, 37 nM, 111 nM, 333 nM, 1 µM and 3 µM. Right graph, chromatogram of CusA binding and elution from IMAC. Continuous line: Zn^2+^ and dashed line: Ni^2+^. FT = Flow-through, EDTA = EDTA elution.

**Figure 5:**
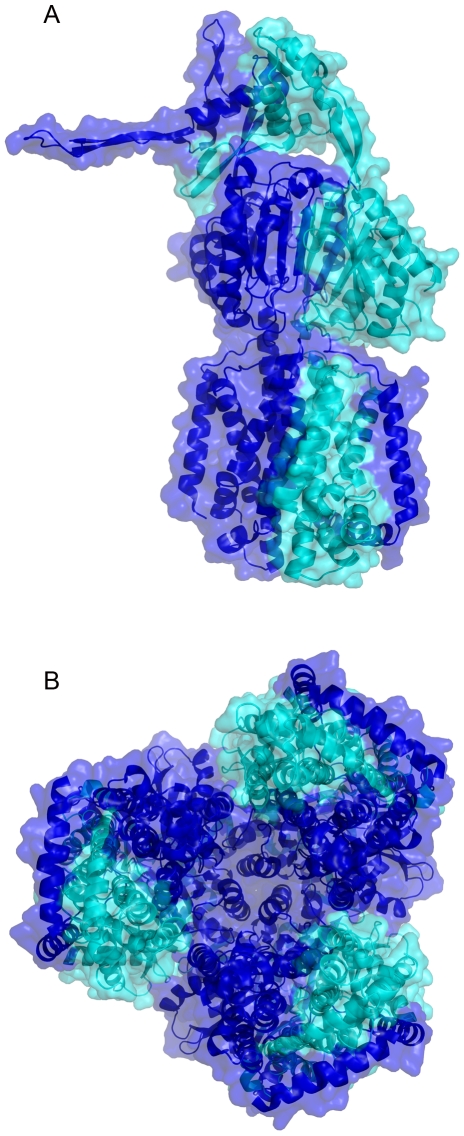
Representation of proteolytic fragments of CusA projected on the AcrB structure. Panel A, the ribbon diagram and the surface of the AcrB monomer is represented in two colours: blue from the N-terminus to residue 612 (equivalent to residue 610 of CusA) and cyan from 613 to the C-terminus. Panel B, the same representation of the AcrB trimer as in panel A highlights the compacity of the region defined by residues 1 to 612 and its importance for the trimer.

The effect of classical detergents with a C_12_ alkyl chain: C_12_E_8_ and lauryldimethylamineoxyde (C_12_DAO); lipids; and novel surfactants: peptergents [13]–[17], amphipols [18], or fluorinated surfactants [19]–[21], on the presence of flexible elements in CusA was investigated (fig. 4A). None of the tested compounds was able to protect the full-length protein from proteolysis. Nonetheless, amphipol, A8–35, and fluorinated surfactant, C_8_FTac_5_ (fig. 4A–C_8_FTac_5_), allowed the stabilisation of the 65 kDa CusA fragment previously observed in C_12_M. In the case of C_8_FTac_5_, after enrichment by gel filtration, mass spectrometry and N-terminal sequencing revealed the presence of 2 main fragments, spanning residues 1–606 and 1–610. CusA and AcrB sequence alignment indicated that these fragments probably resulted from a cleavage in the beginning of the second large periplasmic domain of CusA (fig. 5) and thus corresponded to 7 TM helices. 300 sparse crystallisation conditions directly tested on these CusA-C_8_FTac_5_ purified fragments did not lead to crystals.

#### Divalent cations strategy

Little is known about the substrate specificity of CusA and the transport mechanism by this protein. CusA is supposed to export Cu^+^ and Ag^+^ through the *E coli* inner membrane [5], [22]. Owing to the difficulty to manipulate Cu^+^, the effect of other cations, mainly divalent, was examined on the presence of flexible elements in CusA.

The effect of the addition of 1 mM Ag^+^, Ni^2+^, Cu^2+^, Zn^2+^, Cd^2+^ and Co^2+^ was checked on the limited proteolysis of C_12_M-solubilized CusA. Ag^+^ had no effect (not shown). Co^2+^ (not shown) and Cu^2+^ (fig. 4B) had a slight effect. The major protection was obtained with Ni^2+^, Zn^2+^ (fig. 4B), and Cd^2+^ (not shown). Ni^2+^, Cd^2+^ and Zn^2+^ prevented the chymotrypsinolysis of the full-length protein for at least 2 h to 3 h (fig. 4B). Moreover, Zn^2+^ was the only cation able to prevent trypsinolysis (fig. 4B). The Zn^2+^ effect was also demonstrated for CusA solubilized in C_12_E_8_ and C_12_DAO (not shown). To demonstrate that Zn^2+^ concentrations up to 1 mM do not inhibit the proteolytic activity of trypsin and chymotrypsin, a control experiment was run with p47phox [23] a protein without any relation with zinc ions. As expected, limited proteolysis of p47phox was identical with or without Zn^2+^ and with or without C_12_M (fig. 4C).

SPR experiments and IMAC were carried out to demonstrate that the protecting effect of divalent cations on CusA occurs *via* a direct binding. NTA sensor chips were used to immobilise Ni^2+^, the only divalent cation usable on these sensor chips. A very specific (from 4 nM), dose-dependent and pH-independent binding of CusA-C_12_M on Ni-NTA sensorchips was observed (fig. 4D, left panel). Negative control with BSA in the same condition lead only to negligible signal and positive control with the copper binding protein CopH has been previously published in [24]. The binding to Ni^2+^ but also to Zn^2+^ was confirmed on IMAC. CusA was retained on this column and specifically eluted with EDTA as a metal chelator (fig. 4D, right panel).

To go further, crystallisation assays were run in the presence of increasing Zn^2+^ concentration either in C_12_M and C_12_E_8_. Optimised conditions allowed the observation of interesting granules, which became more angular and crystalline when Zn^2+^ was increased from 100 µM to 5 mM. These objects showed diffraction patterns with only low-resolution rings between 50 and 20 Å (not shown).

## Discussion

Up to now, less than 200 unique membrane protein structures have been deposited in the PDB compared to thousands of soluble proteins. These numbers reflect the difficulty to crystallise and solve the structure of a membrane protein. In fact, one major point arises from the low stability of membrane proteins in solution, *i.e.* extracted from their natural lipid bilayer.

Limited proteolysis has been widely used to assess the presence of flexible loops in soluble proteins, and therefore to identify specific domains suitable for crystallisation [12]. More recently, several membrane protein structures have been obtained thanks to sequence optimisation using this method. For instance, deletion of only 4 residues of Glycerol-3-phosphate transporter resulted in better ordered-crystals [11], and insertion of lysozyme sequence to a flexible loop of β2-adrenergic receptor allowed stabilisation and crystallisation of this G-protein coupled receptor (GPCR) [25]. Limited proteolysis was described in the present work as a useful and simple method to assess the presence of flexible elements in a membrane protein in solution, in order to either directly crystallise it, identify a crystallisable-core region or screen for stabilising conditions.

Regarding the strong efforts made by many laboratories to solve the structure of other proteins of the RND family, we carried on the comparison between AcrB and CusA behaviours. Some differences have been previously pointed out by Stroebel et al.[10]. The present study extensively used limited proteolysis to go further in the comparison and find out new clues to favour crystallisation of RND proteins.

Over 25 AcrB structures have been deposited in the PDB, demonstrating the ability of this protein to crystallise. In the present study, AcrB crystallised in 5% of the tested conditions (fig. 2A) and AcrB crystallogenesis seems very robust. For instance, AcrB still crystallised at concentrations of C_12_M a hundred times higher than in the classical condition. Limited proteolysis assays demonstrated that AcrB has a very rigid core. This probably explains its capacity to crystallise. Indeed, rigidity is often required to obtain well-diffracting crystals. On the other hand, CusA did not crystallise in C_12_M, the most frequently used detergent in crystallisation. CusA in C_12_M is also highly dynamic, explaining why AcrB and CusA have opposite crystallisation behaviours and diverging proteolytic profiles that cannot be explained by a higher cleavage site number in CusA sequence.

If well-defined detergents are routinely used for crystallisation, novel surfactants have been developed to circumvent this stability problem. Amongst these compounds, amphipols or fluorinated surfactants have proved their efficiency on different membrane proteins such as bacteriorhodopsin, cytochrome *b*_6_ *f* or GPCRs [19], [21], [26]. Nonetheless, so far these compounds have only led to crystals unsuitable for structure resolution. Several recent membrane protein crystallisation successes have been obtained by detergent screening [11], [27]. Thus, fourteen different detergents were tested on CusA but crystals were only obtained with C_12_E_8_ (fig. 2C). However, their diffraction corresponded most likely to detergent crystals.

Although limited proteolysis experiments showed that CusA presents an important number of flexible elements and that the identification of a rigid domain is difficult, trypsin or chymotrypsin digestion in the presence of C_12_M led to 30 to 40 kDa CusA fragments starting around residues 280 and 610 as identified by N-terminal sequencing. However, the complexity of the proteolysis mixture in C_12_M greatly complicates the precise identification of each fragment, and the only clear accessible region in different surfactants is located around residue 610. Among all the surfactants that were screened, the best results have been obtained in C_8_FTac_5_ (and amphipol in a lower extent). This surfactant significantly stabilised the 65 kDa CusA fragments released by proteolysis (fig. 4A), which correspond to two major products, CusA 1–606 and CusA 1–610 (fragments released represented in fig. 5). Although the 600–610 CusA region seems particularly flexible, the localisation of these residues on the AcrB structure based on sequence alignment did not highlight a long flexible loop (fig. 1). However, the accessibility of this site by the protease is possible from the periplasmic side (fig. 1C), which is facilitated by CusA low compacity compared to AcrB. Moreover, it is interesting to notice that, by homology with the AcrB structure, the 65 kDa CusA fragments form the inner compact core of the structure (excepting two helices), which corresponds to the whole trimer interface (fig. 5). However it is difficult to speculate about the biological relevance of the structure of this CusA-truncated form and solving its structure would be questionable.

The present study completes the work performed by Stroebel *et al.* [10], and therefore allows a more detailed understanding of the behaviour differences between AcrB and CusA. Stroebel *et al.* [10] proposed that high C_12_M and lipids present in CusA preparation prevented its crystallisation. In the light of the present study, this last point seems negligible to explain CusA non-crystallisation. Our results clearly show that CusA high flexibility appears as a crucial drawback for crystallisation. Thus, defining a locked-form or -construct of CusA could constitute a first step towards its crystallisation.

Addition of substrates or inhibitors that locked the protein conformation has been successful in some cases. For instance, the crystallisation of the bacterial Zn^2+^-transporter Yiip was tested in the presence of several heavy metal cations and sufficiently ordered crystals were obtained only in the presence of 5 mM Zn^2+^ [28]. In this study, the best way to favour CusA crystallisation was the addition of its transported substrates or analogs. Indeed, the number of CusA flexible elements was strongly decreased in the presence of different cations: Zn^2+^, Cd^2+^ and Ni^2+^ (fig. 4B), that probably act by stabilizing the three-dimensional structure of the protein. This effect was due to specific binding of these divalent cations to CusA, as confirmed by SPR measurements and by IMAC retention (fig. 4D). pH drop had no effect on CusA binding to Ni^2+^, confirming that the interaction is not mediated by histidine but rather by methionine as it has been previously proposed [5]. These experiments were also the first demonstration of *in vitro* binding of heavy metal cations to CusA. Moreover, it is interesting to notice that similar observations have been made with CzcA, another HME-RND [29]. CzcA appears reluctant to crystallization and highly dynamic in C_12_M, but Zn^2+^, its preferred substrate [29], stabilises the structure of the protein.

As Zn^2+^ had the strongest protective effect on CusA proteolysis, crystallisation assays were run in the presence of zinc. This ion had an effect on CusA in C_12_M, C_12_E_8_ and C_12_DAO. Crystallisation assays with increasing Zn^2+^concentration were tested to corroborate the decrease of proteolysis with the capacity to crystallise. CusA crystallisation trials in C_12_E_8_ gave the most interesting hits. Granules were observed, comparable to those obtained with AcrB near crystallisation conditions. The presence of these crystalline objects was correlated with the increase of Zn^2+^, and showed that the higher the concentration of zinc, the better and more angular these CusA objects. The more angular objects, obtained in the presence of 1 to 5 mM Zn^2+^, diffracted with spots on rings at low resolution. This can be considered as the first step towards highly ordered three-dimensional crystals of CusA.

In summary, comparison between AcrB and CusA strongly supports the fact that the high flexibility of CusA in C_12_M hampers its crystallisation. The most interesting clue to obtain a crystallisable-form of CusA is certainly the addition of heavy metal cations, especially Zn^2+^, which allowed the appearance of the first CusA crystalline objects. The limited proteolysis method described here could certainly be considered for many other membrane proteins, in order to engineer the protein, by removing flexible loops, or to assess the stabilising effect of additives, amphiphile or ligand, to favour the protein crystallisation.

## Materials and Methods

### Protein purification

AcrB overexpression vector was kindly provided by KM Pos. AcrB was purified as described in [10].

Description of CusA overexpression vectors can be found in [5]. CusA was overexpressed in the *E. coli* strain C43(DE3) as described in [5]. Cells were disrupted by two passages through a French press. After low speed centrifugation, membranes were pelleted by ultracentrifugation (1 h30, 150000 g, 4°C, Beckman Optima LE-80K, rotor 45Ti), resuspended in 0.1 M Tris-HCl pH 8, 0.5 M NaCl, 1 mM EDTA at 20 mg of protein per ml and stored at −80°C. For CusA purification, membrane proteins were solubilised in 1% C_12_M, 50 mM Tris-HCl pH 8, 0.25 M NaCl, 10% glycerol, complete protease inhibitors (Roche) for 1 h at 4°C. After ultracentrifugation for 1 h at 100000 g and 4°C (Beckman Optima LE-80K, SW41 rotor), the supernatant was diluted 1.5 times in 0.1% C_12_M, 50 mM Tris-HCl pH 8, 0.1 M NaCl, 10% glycerol, and mixed with streptactin resin (IBA). After 2 to 4 h of incubation, the resin was packed into a column and washed with increasing NaCl concentration: 25 ml of 0.25 M, 0.5 M and 1 M successively in the same buffer except that C_12_M concentration was decreased down to 0.04%. CusA was eluted in 0.04% C_12_M, 50 mM Tris-HCl pH 8, 0.1 M NaCl, 10% glycerol, 2 mM desthiobiotin. Before crystallisation or limited proteolysis assays the buffer was exchanged by cycles of concentration and dilution in 0.04% C_12_M, 20 mM Tris-HCl pH 8, 0.1 M NaCl on an Amicon concentrator equipped with a 50 kDa cut-off membrane.

For exchange of detergent, CusA bound to the streptactin resin was washed with buffers containing the detergents or surfactants listed in table 1. The final amphiphile concentration was 4 cmc (table 1) in all cases except for amphipols. For exchanging C_12_M to amphipols (A8–35), CusA purified in C_12_M was incubated for 1 hour at 4°C at a ratio of 4 g of amphipols per g of pure CusA. Bio-beads (Biorad) were added to the mixture to adsorb C_12_M and removed by centrifugation after overnight incubation at 4°C.

Protein was concentrated on Amicon concentrators with a 50 kDa cut-off. Protein concentration was estimated from the following theoretical extinction coefficients at 280 nm: 91000 M^−1^cm^−1^ for AcrB, 157000 M^−1^cm^−1^ for CusA.

### Limited proteolysis assays and analysis

Pure protein (CusA or AcrB) at 1 mg/ml was mixed with proteases (chymotrypsin, trypsin, elastase, subtilysin, thermolysin or papain) at the desired weight-to-weight ratio as indicated in the legends of the concerned figures. The proteolysis was started when the protease was added to the protein solution, and the kinetic was stopped by addition of SDS-page loading buffer and freezing at −20°C.

Limited proteolysis was evidenced by SDS-PAGE (8% polyacrylamide). The gels were stained with coomassie blue. CusA proteolytic fragments were identified by N-terminal sequencing and mass spectrometry.

### Surface plasmon resonance (SPR) measurements

SPR experiments were performed on a BIAcore 3000 apparatus using nitrilotriacetic acid (NTA) sensor chip (GE Healthcare). Increasing concentrations of CusA in 0.04% C_12_M, 10 mM Hepes pH 8, 0.1 M NaCl and 500 µM EDTA were injected on a Ni-loaded NTA flow cell, during 3 minutes at 20 µl/min followed by 10 minutes of dissociation. A NTA flow cell was run in parallel in the same conditions as a blank. Between every CusA concentration, flow cells were washed out with 0.3 M EDTA (2×2 min) and 10 mM HCl (1 min). Each binding curve was obtained by double-subtraction [30].

### Immobilised Metal ion Adsorption Chromatography (IMAC)

500 µg of pure CusA in C_12_M was injected onto a Ni^2+^ or Zn^2+^-loaded chelate Hitrap column (GE Healthcare) at a flow rate of 0.4 ml/min. The column was then washed with 10 column volumes of 20 mM Hepes pH 8, 0.1 M NaCl, 0.04% C_12_M. Then, CusA was eluted with 10 column volumes of the same buffer containing 50 mM EDTA.

### Crystallisation experiments

AcrB in C_12_M was concentrated around 10–20 mg/ml. CusA concentration in the different detergents tested is indicated in table 1. Initial screens were performed in 96 well-plate hanging-nanodrops. For a 100 µl reservoir, the drops were made of 100 nl of protein plus 100 nl of reservoir. All QIAgen commercial screens were used. Manual optimisation was carried out with drops of 1 µl of protein plus 1 µl of reservoir, for a 500 µl reservoir.

For diffraction tests, crystals or crystalline objects were harvested and frozen in liquid nitrogen. Diffraction data were collected at 100 K on ID14eh2, ID14eh4 and ID23eh2 beamlines at the ESRF Grenoble.
